# USP5 Sustains the Proliferation of Glioblastoma Through Stabilization of CyclinD1

**DOI:** 10.3389/fphar.2021.720307

**Published:** 2021-08-16

**Authors:** Gen Li, Tianquan Yang, Yanling Chen, Jianping Bao, Di Wu, Xiaohan Hu, Chenxi Feng, Lixiao Xu, Mei Li, Gang Li, Meifang Jin, Yunyun Xu, Rui Zhang, Guanghui Qian, Jian Pan

**Affiliations:** ^1^ Institute of Pediatric Research, Children’s Hospital of Soochow University, Suzhou, China; ^2^ Laboratory of Molecular Neuropathology, College of Pharmaceutical Sciences, Soochow University, Suzhou, China; ^3^ Department of Neurosurgery, Children’s Hospital of Soochow University, Suzhou, China; ^4^ School of Basic Medicine and Biological Sciences, Soochow University, Suzhou, China; ^5^ Department of Neonatology, Children’s Hospital of Soochow University, Suzhou, China; ^6^ Clinical Pediatrics School, Soochow University, Suzhou, China

**Keywords:** glioblastoma multiforme, USP5, CyclinD1, deubiquitination, cell cycle

## Abstract

Glioblastoma multiforme (GBM) is one of the most malignant primary tumors in humans. Despite standard therapeutic strategy with tumor resection combined with radiochemotherapy, the prognosis remains disappointed. Recently, deubiquitinating enzymes (DUBs) has been reported as potential cancer therapy targets due to their multifunctions involved in the regulation of tumorigenesis, cell cycle, apoptosis, and autophagy. In this study, we found that knockdown of ubiquitin specific protease (USP5), a family member of DUB, could significantly suppress GBM cell line U251 and DBTRG-05MG proliferation and colony formation by inducing cell cycle G1/S arrest, which was correlated with downregulation of CyclinD1 protein level. CyclinD1 had been reported to play a critical role in the tumorigenesis and development of GBM *via* regulating cell cycle transition. Overexpression of USP5 could significantly extend the half-life of CyclinD1, while knockdown of USP5 decreased the protein level of CyclinD1, which could be restored by proteasome inhibitor MG-132. Indeed, USP5 was found to directly interact with CyclinD1, and decrease its K48-linked polyubiquitination level. Furthermore, knockdown of USP5 in U251 cells remarkably inhibited tumor growth *in vivo*. Taken together, these findings demonstrate that USP5 plays a critical role in tumorigenesis and progression of GBM by stabilizing CyclinD1 protein. Targeting USP5 could be a potential therapeutic strategy for GBM.

## Introduction

Glioblastoma multiforme (GBM) is one of the most malignant primary tumors originated by neuroglial stem or progenitor cells, which may occur at any age (Weller et al., 2015). There are approximately 13,000 new cases diagnosed in the USA every year (Reardon and Mitchell, 2017). Despite the development of current GBM therapeutic strategies includes surgical resection, chemotherapy, and radiotherapy, or a combination of these treatments, the prognosis remains gloomy (Ostrom et al., 2014). The average survival of GBM patients is about 14 months (Stupp et al., 2009), and the 5-years-survival rates are less than 9.8% (Stupp et al., 2009; Carlsson et al., 2014). Hence, identification of new molecules involved in GBM tumorigenesis and progression is urgent for the development of more effective therapeutic strategies against this malignant tumor.

Among post-translational modifications, ubiquitination plays a critical role by regulating a wide range of cellular biological progress, such as cell proliferation, cell cycle progression, apoptosis, inflammatory response, and DNA-damage repair (Hoeller and Dikic, 2009; Pal et al., 2014; Liu et al., 2017a; Yan et al., 2017). Ubiquitylation could be reversed by deubiquitinating enzymes (DUBs). DUBs are a large group of proteases which could mediate the removal of ubiquitin molecules from target proteins (Mevissen and Komander, 2017; Young et al., 2019). The human genome encodes approximately 115 DUBs, including six subclasses: ubiquitin specific proteases (USPs), ovarian tumor proteases (OTUs), ubiquitin carboxyterminal hydrolases (UCHs), Machado-Joseph disease proteases (MJDs), JAB1/MPN/Mov34 metalloenzymes (JAMMs), and motif interacting with Ub-containing novel DUB family (MINDY) (Clague et al., 2013; Abdul Rehman et al., 2016; Weisberg et al., 2017). Recently, increasing investigations have suggested that USP family has critical function in tumor cell cycle (Kaistha et al., 2017), apoptosis (Wang et al., 2017), metastasis (Meng et al., 2019), and other biological progress (Ling et al., 2017; Gadotti and Zamponi, 2018; Sun et al., 2020).

In recent years, DUBs have exerted critical roles in GBM *via* targeting multiple key proteins involved in the regulation of tumorigenesis, cell cycle apoptosis, and autophagy. Previous studies had reported that USP1, USP8, USP11, and USP28 were indispensable for GBM growth via increasing the protein stability of ID1, FLIPS, PML, and c-Myc, respectively (Lee et al., 2010; Panner et al., 2010; Guo et al., 2013; Wu et al., 2014). Moreover, USP8, USP12, USP22 could promote the radioresistance of GBM stem cells by stabilizing Hedgehog pathways, Notch, and Sirt1, respectively (Chang et al., 2009; Wang et al., 2010; Santoni et al., 2013). Furthermore, USP22 promoted GBM chemoresistance to temozolomide by stabilizing ZEB1 (Siebzehnrubl et al., 2013). Thus, it would be of great importance to uncover more DUB members involved in GBM tumorigenesis and progression, which could be potential targets for GBM clinical therapy.

Ubiquitin specific protease 5 (USP5) belongs to the USP subfamily, which is located at chromosome 12p13, and encodes a 93.3 KDa protein (Wilkinson et al., 1995; Ansari-Lari et al., 1996). According to the current research, USP5 is reported to be involved in multiple biological progress, such as inflammatory response (Liu et al., 2018) and DNA damage repair (Nakajima et al., 2014). Furthermore, USP5 is also found to contribute to the tumorigenesis and progression of many malignancies, including neuroblastoma (Cheung et al., 2021), hepatocellular carcinoma (Liu et al., 2017b; Meng et al., 2019), multiple myeloma (Mollaoglu et al., 2017; Wu et al., 2020), pancreatic carcinoma (Kaistha et al., 2017; Li et al., 2017; Lian et al., 2020), ovarian cancer (Kim et al., 2018; Du et al., 2019), colorectal cancer (Xu et al., 2019), and non-small cell lung cancer (Ma et al., 2018; Xue et al., 2020). According to the GEPIA2 database (http://gepia2.cancer-pku.cn), compared with normal brain tissues, the mRNA level of USP5 in GBM tissues shows an increasing trend to some extent, indicating that USP5 may facilitate GBM tumorigenesis, and progression.

## Materials and Methods

### Cell Culture, Antibodies, and Chemicals

The human GBM cell lines U251 and DBTRG-05MG, and human embryonic kidney cell line (293T) were purchased from the American Type Culture Collection. These cells were cultured in Dulbecco’s modified Eagle’s medium (DMEM) supplemented with 10% fetal bovine serum (FBS), 100 μg/ml of penicillin and 100 units/ml of streptomycin. The primary antibodies against USP5 (ab154170), CyclinD1 (ab134175), CyclinE1 (ab33911), CDK2 (ab32147), CDK4 (ab108357), Ki67 (ab16667), PCNA (ab18197), Ubiquitin (linkage-specific K48, ab140601) were purchased from Abcam (USA), CDK6 (13,331), and β-Actin (3,700) were purchased from Cell Signal Technology (USA). MG-132 (S2619) was purchased from Selleckchem (USA). Doxycycline hyclate (DOX, D9891) and Cycloheximide (CHX,239763) were purchased from Sigma (USA).

### Stable Cell Establishment for USP5 Overexpression

Lentiviral plasmid expressing USP5 with 3 × flag tag was generated by GENEWIZ (China). To generate lentiviral particles, HEK293T cells at 80% confluence in a 10 cm dish were co-transfected with 8 μg target plasmid, 6 μg psPAX2 (addgene, United States), 2 μg pMD2.G (addgene, United States) using PEI (Sigma, United States) as a gene delivery carrier. After being washed and refreshed with the DMEM medium, cells were further cultured for 30 h. The lentiviral particle-enriched supernatant was harvested, filtered, and stored frozen at −80°C. After titration, these lentiviral particles were applied to infect U251 and DBTRG-05MG cells for 96 h, and using 2 mg/ml puromycin to establish stable expression cells.

### Stable Cell Establishment for USP5 Knockdown

U251 and DBTRG-05MG cell lines stably expressing USP5-specific shRNA or scrambled shRNA control were constructed and lentivirus particles were packaged by Genechem (China). U251 and DBTRG-05MG were infected with serial dilutions of lentiviral supernatant and selected for using 2 mg/ml puromycin for 2 weeks. The expression of shRNA should be induced by 2 ug/mL DOX for 3 days. The human USP5 shRNA targeting sequences are listed as follows. The targeting sequence for USP5-shRNA#1 was 5′- TTG​CCT​TCA​TTA​GTC​ACA​T-3′; the targeting sequence for USP5-shRNA#2 was 5′- TAG​ACA​TGA​ACC​AGC​GGA​T-3′; the targeting sequence for USP5-shRNA#3 was 5′- CGA​GGA​GAA​GTT​TGA​ATT​A-3′; the targeting sequences for scrambled shRNA was 5′- TTC​TCC​GAA​CGT​GTC​ACG​T-3′.

### Cell Proliferation and Colony Formation Assay

Cell proliferation assays were performed by CCK-8 assay. Cells (2 × 10^3^/well) were seeded into 96-well plates with 2 ug/mL DOX added into the cultural medium. 10 µL CCK-8 solution (DOJINDO, Japan)/100 uL medium was added and incubated for an additional 2 h. Then, the absorbance at 450 nm was measured using a Microplate Absorbance Reader (Bio-Rad, United States). As to colony formation assay, tumor cells (1 × 10^3^/well) were plated into 6-well plates with 2 ug/mL DOX added into the cultural medium and incubated for 14 days. Cell colonies were fixed with 4% formaldehyde for 10 min and later stained with 0.1% crystal violet dye for 5 min.

### Cell Migration Assay

Cells were plated into 6-well dishes and treated with DOX for 3 days. Then cells were scratched in the center of the well and continuously cultured with 1% FBS medium. Wound images were photographed every 12 h using a light microscope (Nikon, Japan).

### Edu Staining Analysis

Edu staining was performed using BeyoClick™ EdU Cell Proliferation Kit with Alexa Fluor 488 (Beyotime, China) according to the manufacturer’s protocol. Briefly, cells were incubated with 10 μM Edu for 2 h at 37°C, and then fixed with 4% paraformaldehyde for 10 min, and blocked with 3% BSA in 0.1% PBS-Triton X-100 for another 1 h. The cells were incubated with the Click Reaction Mixture for 30 min at room temperature in a dark place and then incubated with DAPI for 5 min.

### Flow Cytometric Analysis

Cell cycle and apoptosis were assayed using Cycle and Apoptosis Analysis Kit (Beyotime, China) according to the manufacturer’s protocol. Briefly, cells were collected by centrifuging at 1,000 rpm at 4°C for 5 min, cell pellets were washed twice with cold PBS (Procell, China). For apoptosis, cells were labelled with propidium-iodide mixture and Annexin V-FITC and subsequently measured by the flow cytometer GALLIOS (Beckman Coulter, United States). For cell cycle, cells were re-suspended and fixed with ice-cold ethanol (70%) overnight, and then labelled with propidium-iodide mixture and subsequently measured by the flow cytometer GALLIOS. For data evaluation, the software FlowJo ver. 7.6.5 (Tree Star Inc., Oregon, United States) was used.

### RNA Extraction, cDNA Synthesis, and qRT-PCR

Total RNA was extracted from tumor cells using TRIzol reagent (Invitrogen, United States), and cDNA was then synthesized with 5 × all-in-one RT MasterMix (abm, Canada) according to the manufacturer’s protocol. Quantitative real-time Reverse Transcription PCR (qRT-PCR) was conducted with 2 × SYBR Green qPCR MasterMix (Bimake, United States). The relative mRNA expression was calculated after normalization to GAPDH. The primer sequences are as following: USP5, 5′CGG​ATT​TGA​CCT​TAG​CG-3′ (Forward) and 5′-CTG​CCA​TCG​AAG​TAG​CG-3′ (Reverse), GAPDH, 5′-ATC​ATC​CCT​GCC​TCT​ACT​GG-3′ (Forward) and 5′-CCC​TCC​GAC​GCC​TGC​TTC​AC-3′ (Reverse), CyclinD1, 5′-CCC​TCG​GTG​TCC​TAC​TTC​A-3′ (Forward) and 5′- CTC​CTC​GCA​CTT​CTG​TTC​CT-3′ (Reverse), CyclinE1, 5′- CAG​CCT​TGG​GAC​AAT​AAT​GC-3′ (Forward) and 5′- TTG​CAC​GTT​GAG​TTT​GGG​TA -3′ (Reverse), CDK2, 5′- CAG​GAT​GTG​ACC​AAG​CCA​GT-3′ (Forward) and 5′- TGA​GTC​CAA​ATA​GCC​CAA​GG-3′ (Reverse), CDK4, 5′- ATG​GCT​ACC​TCT​CGA​TAT​GAG​C-3′ (Forward) and 5′- CAT​TGG​GGA​CTC​TCA​CAC​TCT-3′ (Reverse), CDK6, 5′- GTG​AAC​CAG​CCC​AAG​ATG​AC-3′ (Forward) and 5′- TGG​AGG​AAG​ATG​GAG​AGC​AC-3′ (Reverse), PCNA, 5′- GGC​GTG​AAC​CTC​ACC​AGT​AT-3′ (Forward) and 5′- TTC​TCC​TGG​TTT​GGT​GCT​TC-3′ (Reverse).

### Immunoblotting and Immunoprecipitation

For protein extraction, cells were collected by centrifuging at 1,000 rpm at 4°C for 5 min, cell pellets were washed twice with cold PBS (Procell, China) and then re-suspended in appropriate RIPA lysis buffer (Beyotime, China). Protein concentration was detected by Enhanced BCA Protein Assay Kit (Beyotime, China). Western blotting was done by electrophoresing 20 μg proteins on SDS–PAGE and subsequently transferring electrophoretically onto PVDF membranes (Millipore, Germany). Blocking was done in 5% non-fat dry milk in 0.1% TBST for 1 h at room temperature and then incubated with appropriate primary at 4°C overnight. Then the membranes were incubated with HRP-labeled corresponding secondary antibodies for 1 h at room temperature and Super Signal West Dura Extended kit (Thermo Scientific, United States) was used to detect the result. β-actin was used as the internal control.

For immunoprecipitation assay, the supernatants with respective antibodies were incubated on a rotor at 4°C overnight. Then the protein G agarose beads (Millipore, Germany) was added into the mixture and incubated with rotation for an additional 3 h at 4°C. Next, the immunoprecipitates were washed three times with cold washing buffer. Finally, the immunoprecipitates resuspended in loading buffer containing β-mercaptoethanol were denatured by 100°C for 10 min and then were subjected to SDS-PAGE experiment.

### *In Vivo* Procedure for Intracranial GBM Cell Implantation

All animal experiments were performed under the approval of the Animal Care and Use Committee at Children’s Hospital of Soochow University, and the experimental procedures for all mice were performed in accordance with the Regulations for the Administration of Affairs Concerning Experimental Animals approved by the State Council of the People’s Republic of China. (No. ESCU-201700047, ESCU-201700046).

Briefly, eight athymic nude mice were randomized into 2 groups, and anaesthetized with Ketalar and xylazine (10 mg/kg) and stuck into a stereotactic head frame. A 1.5-mm bur hole was trained one metric linear unit anterior to the sutura on the proper hemisphere and a pair of metric linear unit lateral from the midplane. A Hamilton syringe fastened onto the pinnacle frame was injected 5 × 10^5^ U251 in 5 μL PBS into the left striatum of the brain. The skin incision was then closed with 4–0 silk thread. Suitable medications were provided to scale back pain. To induce USP5 knockdown, mice were fed 2 mg/ml doxycycline in drinking water. The tumor tissues were collected after 30 days and prepared for IHC staining. The tumor volume was calculated as πls^2^/^6^, where l represented the long side of the tumor, and s represented the short side of the tumor. For IHC staining, antigen retrieval was performed using 10 mM citrate buffer (pH 6.0) heated in a pressure cooker for 90 s. USP5, CyclinD1, Ki-67, PCNA antibodies were applied overnight at 4°C. Immunostaining was performed by using Envision + System and diaminobenzidine (DAB) visualization (Dako, Carpinteria, CA). Sections were counterstained with hematoxylin and examined by light microscopy.

### Statistics

All data were displayed as means ± standard deviations (SD). Statistical difference between the control and the experimental groups was analyzed by the two-tailed student’s *t*-test. *p* < 0.05 was deemed statistically significant.

## Results

### USP5 was Essential for GBM Cell Proliferation *in vitro*

**FIGURE 1:**
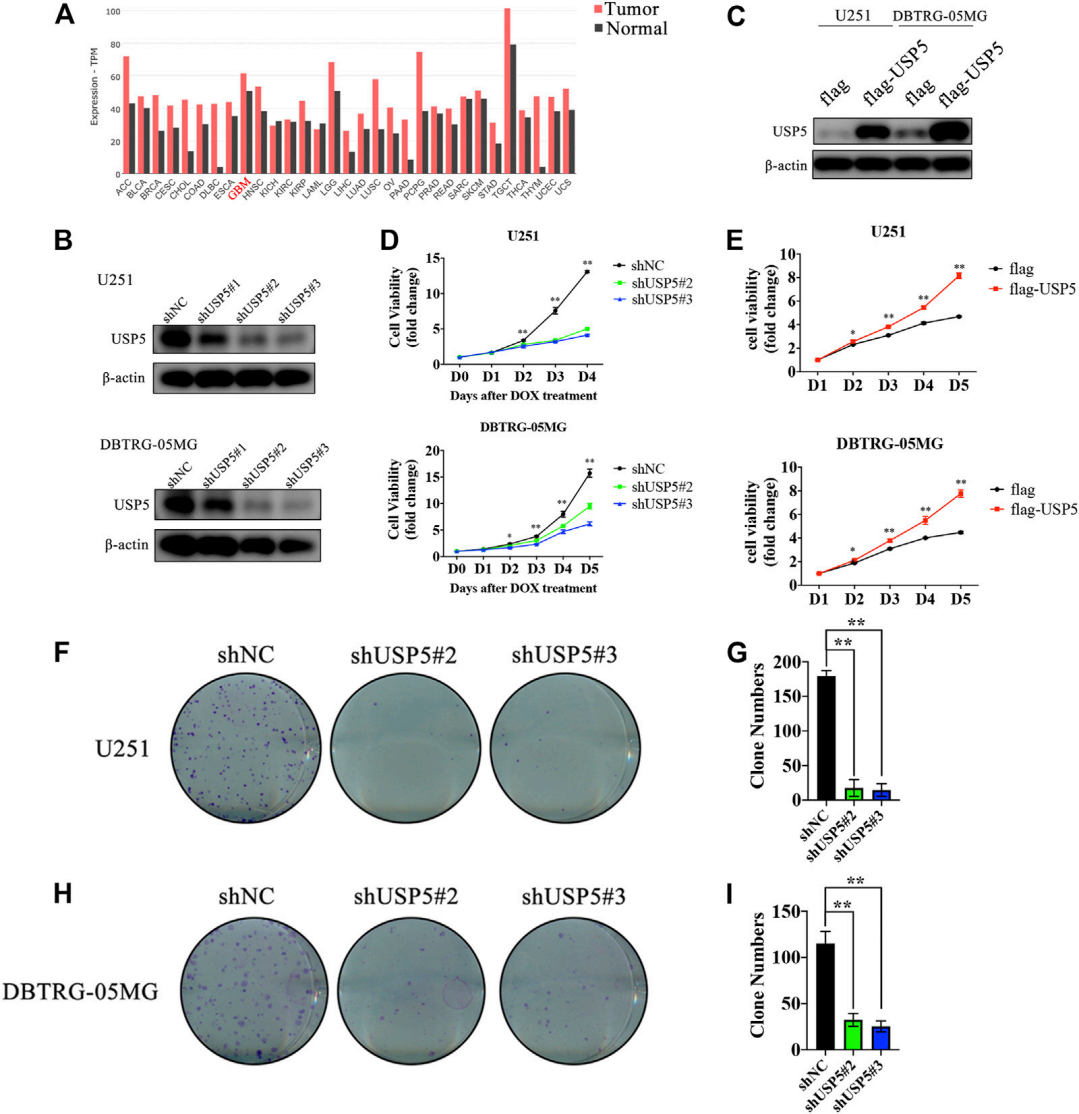
USP5 was closely related with GBM cells proliferation **(A)** USP5 mRNA expression level in a broad range of tumors and relevant normal tissues (generated from GEPIA2: http://gepia2.cancer-pku.cn) **(B)** U251 and DNTRG-05MG cells were infected with USP5 shRNAs (#1, #2, and #3) and control shRNA (shNC) by lentivirus, shRNA expression was induced by 2 ug/mL Doxycycline (DOX) treatment for 72 h. Western blotting analysis was performed to check the knockdown efficiency of USP5 shRNAs **(C)** U251 and DNTRG-05MG cells were infected with USP5 overexpression plasmid with 3 × flag tag (flag-USP5) and empty vector (flag) by lentivirus. Western blotting was performed to check the expression level of USP5 **(D)** Proliferation of U251 and DBTRG-05MG cells expressed shNC or shUSP5 (#2, #3) was detected by CCK-8 assay after DOX treatment at indicated time points **(E)** Proliferation of U251 and DBTRG-05MG cells expressed flag or flag-USP5 was detected by CCK-8 assay at indicated time points **(F–I)** Clone formation levels of U251 and DBTRG-05MG cells expressed shNC or shUSP5 (#2, #3) after DOX treatment for 14 days (Data were presented with mean ± SD of three independent experiments, *p < 0.05, **p < 0.01).

**FIGURE 2:**
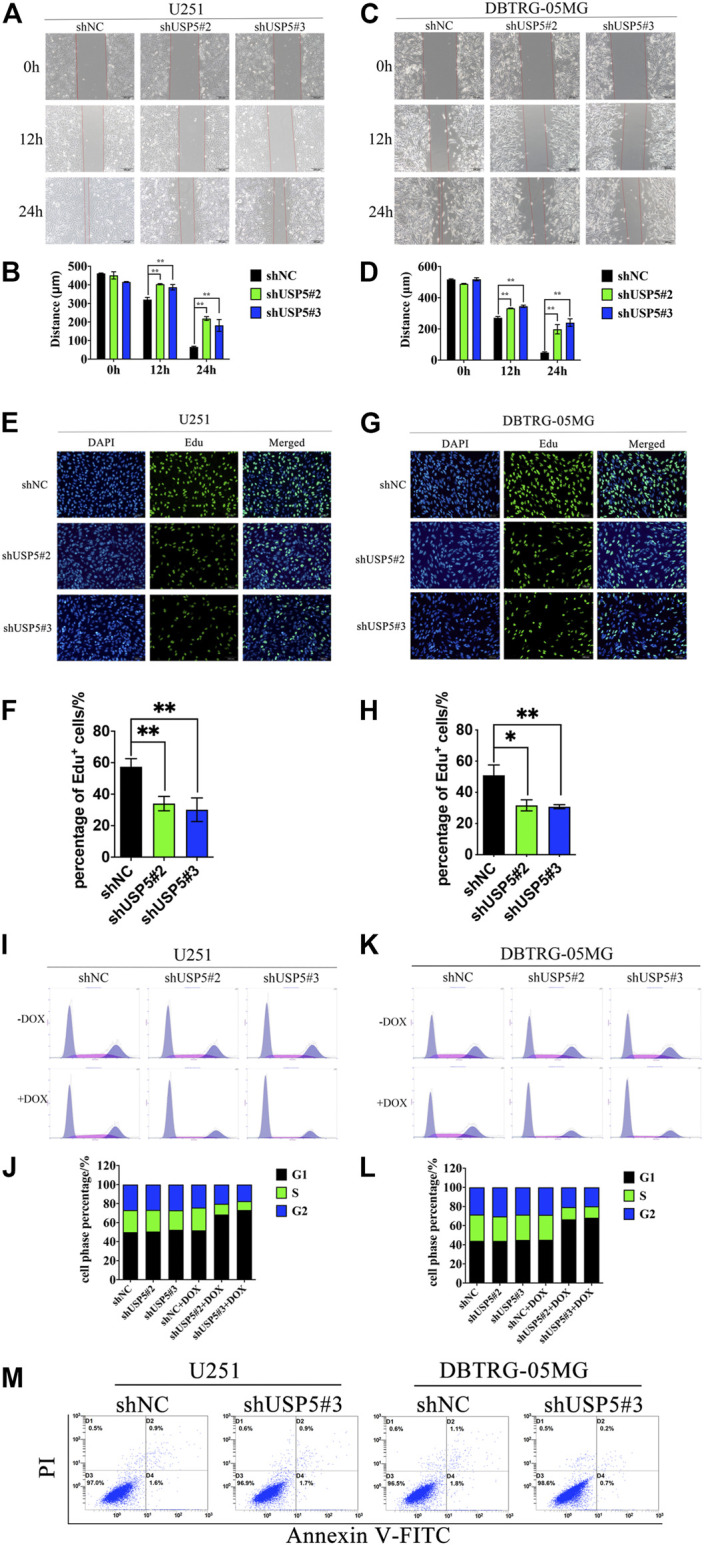
USP5 knockdown inhibited GBM cell migration and induced cell cycle arrest in G1 phase **(A–D)** U251 and DBTRG-05MG cells stably expressed shNC or shUSP5 (#2, #3) were treated with 2 ug/mL DOX for 72 h. Migration of U251 and DBTRG-05MG cells were determined based on a wound-healing assay. Black bar, 200 μm **(E–H)** U251 and DBTRG-05MG cells stably expressed shNC or shUSP5 (#2, #3) were treated with 2 ug/mL Dox for 72 h, then cells were incubated with 10 μM Edu for 2 h. Edu incorporation levels were determined by BeyoClick™ EdU Cell Proliferation Kit with Alexa Fluor 488. White bar, 100 μm **(I–L)** U251 and DBTRG-05MG cells stably expressed shNC or shUSP5 (#2, #3) were treated with 2 ug/mL DOX (+ DOX) or PBS (−DOX) for 72 h. Cell cycle distribution of U251 and DBTRG-05MG cells was assessed by PI staining and flow cytometry analysis. Representative graphs and statistical analysis of percentages at different cell cycle stages are shown **(M)** U251 and DBTRG-05MG cells stably expressed shNC or shUSP5 #3 were treated with 2 ug/mL DOX (+ DOX) or PBS (−DOX) for 72 h. Cell apoptosis of U251 and DBTRG-05MG cells was assessed by PI and Annexin V-FITC staining and flow cytometry analysis (Data were presented with mean ± SD of three independent experiments, *p < 0.05, **p < 0.01).

By searching the GEPIA2 database, USP5 was found to be highly expressed in most cancers, including GBM (Figure 1A). USP5 had been reported to contribute to the tumorigenesis and progression of many malignancies, however, the function of USP5 in GBM is still unknown. Thus, USP5 was knockdown or overexpressed in GBM cell lines U251 and DBTRG-05MG, respectively, *via* lentivirus-mediated plasmid transduction (Figures 2B,C). USP5 knockdown was induced by doxycycline treatment for 72 h. The CCK8 assay showed that knockdown of USP5 significantly suppressed proliferation of U251 and DBTRG-05MG cells, while its overexpression relatively increased cell proliferation (Figures 1D,E). Moreover, USP5 knockdown also remarkably inhibited clone formation of U251 and DBTRG-05MG cells (Figures 1F–I). These results suggested that USP5 was required for GBM cell growth *in vitro*.

### Knockdown of USP5 Suppressed GBM Cell Migration and Cell Cycle Progression

As USP5 could sustain GBM cell proliferation, leading us to further investigate its effect on cell phenotype. Wound healing assay showed that, compared with negative control, knockdown of USP5 could prominently prohibit U251 and DBTRG-05MG migration (Figures 2A–D). Moreover, Edu incorporation assay revealed that U251 and DBTRG-05MG were significantly arrested after USP5 knockdown (Figures 2E–H), indicating that USP5 was critical for GBM cell cycle progression. To confirm this hypothesis, cell cycle was determined by cytometry. As shown in Figures 2I–L, U251 and DBTRG-05MG were observably arrested in the G_1_ phase after USP5 downregulation. Interestingly, cell apoptosis was not induced by USP5 knockdown (Figure 2M). These results indicated that USP5 was important for GBM cell migration and cell cycle progression.

### USP5 Knockdown Decreased CyclinD1 Protein Stability

**FIGURE 3:**
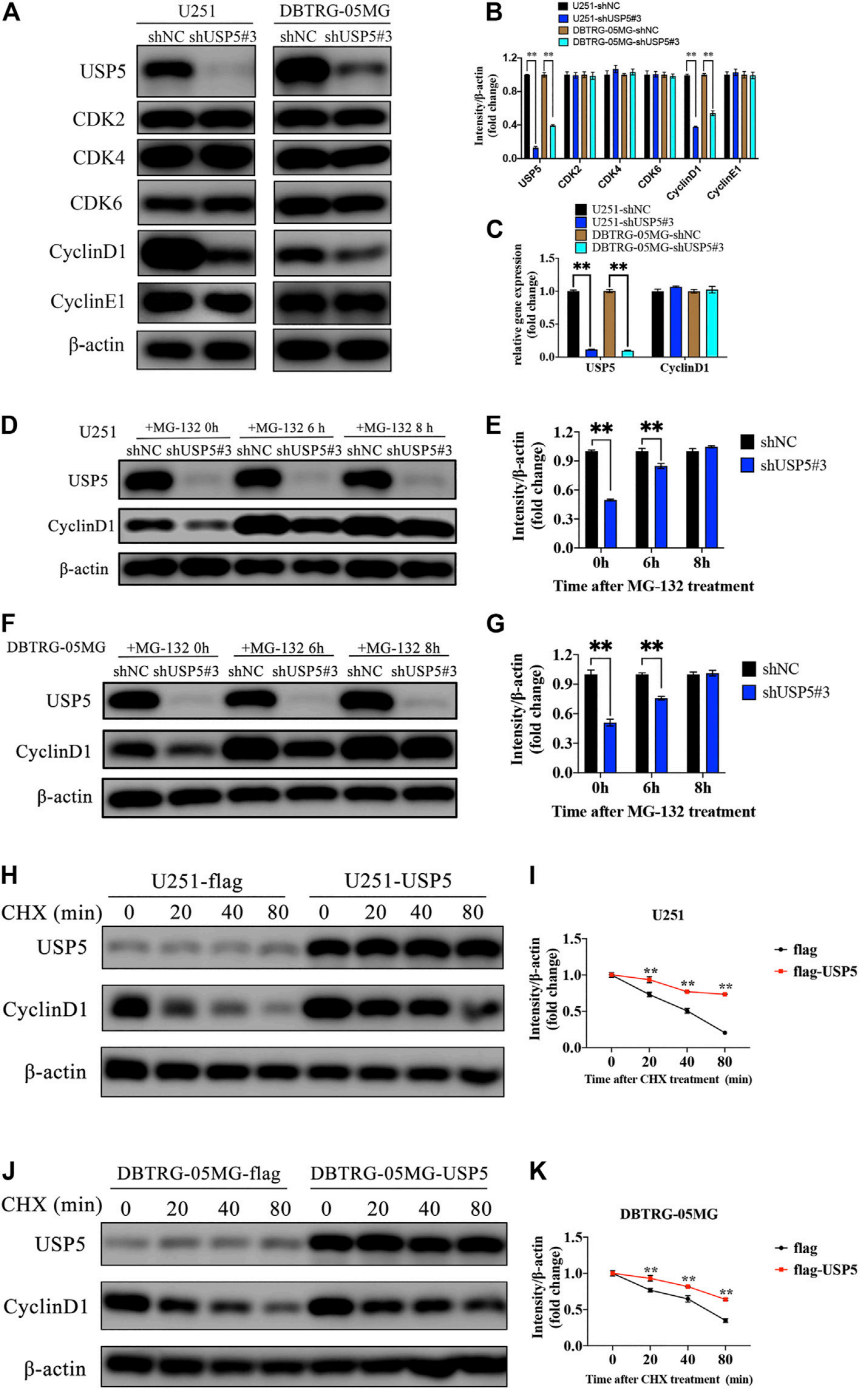
USP5 maintained CyclinD1 stability **(A, B)** Western blotting analysis of USP5, CDK2, CDK4, CDK6, CyclinD1, CyclinE1, and β-actin protein levels in U251 and DBTRG-05MG cells stably expressed shNC or shUSP5#3 treated with 2 ug/mL Dox for 72 h **(C)** Real-time PCR analysis of USP5 and CyclinD1 mRNA levels in U251 and DBTRG-05MG cells stably expressed shNC or shUSP5 #3 treated with 2 ug/mL Dox for 72 h **(D–G)** U251 and DBTRG-05MG cells stably expressed shNC or shUSP5#3 treated with 2 ug/mL Dox for 72 h were incubated with 20 μM MG-132 for indicated time points. Western blotting analysis of USP5, CyclinD1, and β-actin protein levels **(H–K)** U251 and DBTRG-05MG cells stably expressed empty vector (flag) or USP5 overexpression (flag-USP5) were incubated with 100 μg/ml CHX for indicated time points. Western blotting analysis of USP5, CyclinD1, and β-actin protein levels (Data were presented with mean ± SD of three independent experiments, *p < 0.05, **p < 0.01).

The above studies showed that GBM cells were arrested in cell cycle G1 phase after USP5 knockdown, so we wondered whether USP5 regulated key proteins driving cell cycle G1 to S transition, including CDK2, CDK4, CDK6, CyclinD1, and CyclinE1 (Malumbres and Barbacid, 2009; Musgrove et al., 2011). As shown in Figures 3A,B, knockdown of USP5 in U251 and DBTRG-05MG cells specifically decreased CyclinD1 protein level. Interestingly, USP5 knockdown had no effect on CyclinD1 mRNA level (Figure 3C). USP5 is a member of deubiquitinases, which leading us to hypothesize the regulation of USP5 on CyclinD1 protein stabilization. In U251 and DBTRG-05MG cells, the downregulation of CyclinD1 protein followed by USP5 knockdown could be rescued by treatment of proteasome inhibitor MG-132 (Figures 3D–G), which indicated that USP5 could prevent CyclinD1 from proteasome degradation. To confirm this finding, USP5 was overexpressed in U251 and DBTRG-05MG cells, followed by treatment of CHX, an inhibitor of protein synthesis. The results showed that USP5 overexpression prolonged the half-life of CyclinD1 (Figures 3H–K). Taken together, these results suggested that USP5 sustained the stability of CyclinD1.

### USP5 Interacted With CyclinD1 Protein and Decreased Its k48-Linked Polyubiquitination Modification

**FIGURE 4:**
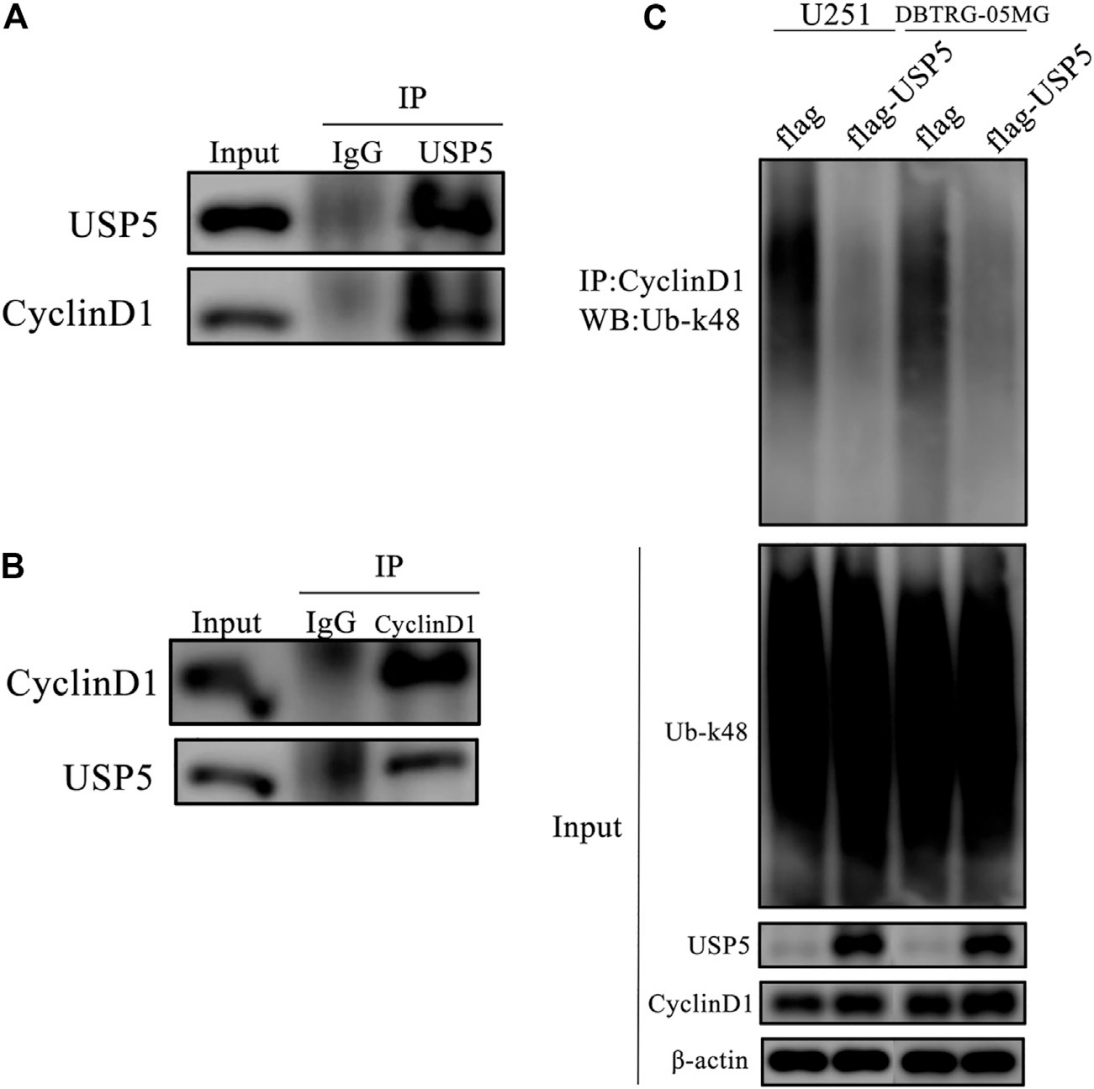
USP5 directly interacted and deubiquitinated and CyclinD1 **(A, B)** USP5 **(A)** or CyclinD1 **(B)** was immunoprecipitated from U251 cells, respectively. Control immunoprecipitations were with nonspecific IgG, followed by western blotting of the precipitated proteins with antibodies for USP5 and CyclinD1 **(C)** CyclinD1 was immunoprecipitated from U251 and DBTRG-05MG cells stably expressed empty vector (flag) or USP5 overexpression (flag-USP5), respectively, followed by western blotting of the precipitated proteins with the antibody specifically for k48-linked ubiquitin (Ub-k48).

According to the above studies, CyclinD1 protein stability was dependent on USP5. Because the core function of USP5 is to prevent protein ubiquitination, and k48-linked polyubiquitination is the main type of modification for protein degradation, so we wondered whether USP5 removed k48-linked polyubiquitination chain from CyclinD1 protein. To confirm this supposition, co-immunoprecipitation assay was performed in U251 cells. As shown in Figures 4A,B, USP5 could interact with CyclinD1. To uncover the effect of USP5 on the ubiquitination level of CyclinD1, USP5 was overexpressed in U251 and DBTRG-05MG cells, respectively. The results showed that forced expression of USP5 noticeably decreased CyclinD1-associated k48-linked polyubiquitination (Figure 3C). Overall, these results indicated that USP5 bound to CyclinD1 and downregulated the k48-linked polyubiquitination level.

### Knockdown of USP5 Inhibited GBM Growth *in vivo*

**FIGURE 5:**
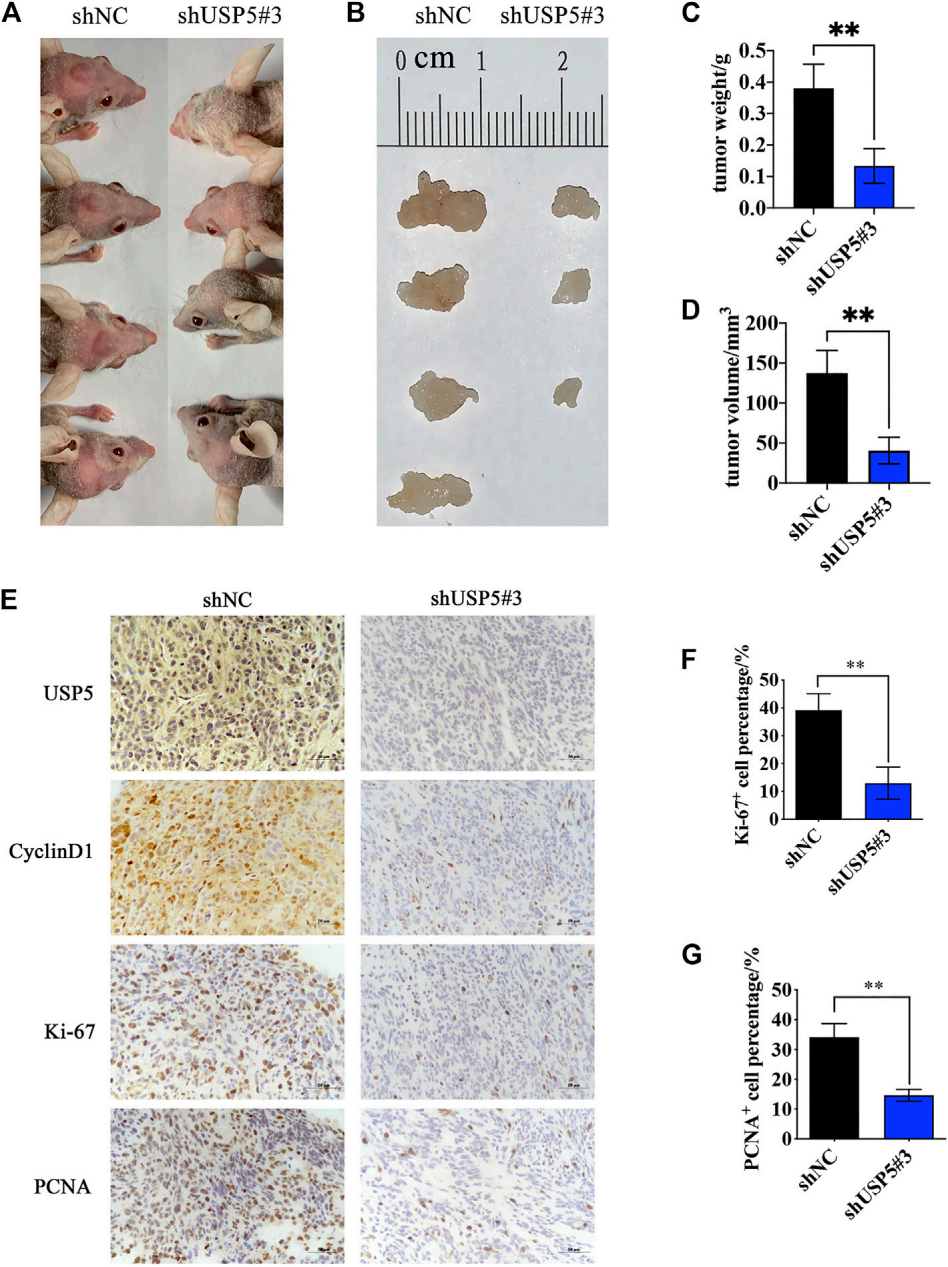
Ablation of USP5 impaired the tumorigenicity of GBM cells *in vivo*. 5 × 10^5^ U251 stably expressed shNC or shUSP5#3 were intracranially injected into nude mice, respectively (*n* = 4). ShRNA expression was induced by 2 mg/ml DOX in drinking water, and tumors were collected 30 days after induction **(A, B)** Photograph of xenograft tumors from shNC or shUSP5#3 mice **(C)** Tumor weight from shNC or shUSP5#3 mice **(D)** Tumor volume from shNC or shUSP5#3 mice **(F)** IHC staining of USP5, CyclinD1, Ki-67, and PCNA in xenograft tumors from shNC or shUSP5#3 mice **(F, G)** Ki-67 or PCNA positive cells count in IHC staining sections of xenograft tumors from shNC or shUSP5#3 mice (Data were presented with mean ± SD of three independent experiments, *p < 0.05, **p < 0.01).

To investigate the function of USP5 for GBM growth *in vivo*, we established orthotopic tumor models by intracranially implanting U251-shNC and U251-shUSP5#3 cells into nude mice. As shown in Figures 5A,B, mice in the control group all developed tumors, while only three quarters in the USP5 knockdown group produced tumors. Moreover, compared with the control group, knockdown of USP5 remarkably decreased tumor sizes (Figures 5C,D). Immunohistochemical staining revealed that the levels of CyclinD1 and proliferation markers Ki-67 and PCNA were subsequently decreased after USP5 knockdown in xenograft sections (Figures 5E–G). These data indicated that USP5 was essential for GBM growth *in vivo* via regulating CyclinD1.

## Discussion

GBM remains one of the most malignant tumors worldwide with disappointed prognosis under current therapeutic strategies. Multiple molecules participated in the tumorigenesis and development of GBM. Hence, there is an urgent need to determine the vital genes concerned in tumor progress, providing promising strategies for precise therapy of patients with GBM. As ubiquitination modifications play a critical role in almost all cellular progresses, especially in cancers (Rape, 2018). Increasing evidences by recent studies indicate that DUBs are also involved in these activities (Harrigan et al., 2018). Our present investigation showed that USP5 was essential for GBM growth both *in vitro* and *in vivo*. We then confirmed the function of USP5 in the migration and cell cycle G1 to S progression of GBM cells. Furthermore, the underlying mechanism of USP5 in regulating GBM cell cycle arrest was clarified that USP5 directly targeted and stabilized CyclinD1 by suppressing its k48-linked polyubiquitination. These results indicated that USP5 could be an effective target for clinical GBM molecular therapy.

The proteins belonging to the USP family have already been reported to play vital roles in a variety of cancer associate progress. USP5 also has been shown to be a tumor suppressor in a number of malignant cancers by regulating different proteins. It had been reported that in GBM cell lines U87 and T98G, co-knockdown of SF2/ASF1 in addition to USP5 inhibited cell proliferation and induced apoptosis *via* regulating hnRNPA1 through its deubiquitinase activity (Vashistha et al., 2020). Here, we reported that USP5 was also critical for GBM cell lines U251 and DBTRG-05MG proliferation via mediating cell cycle G1 to S progression without any alteration on cell apoptosis. These contrasts may be due to the tumor heterogeneity, as different GBM cell lines were used in the two independent investigations. Our data provided another insight into the functionality of USP5 in the tumorigenesis and development of USP5-related GBM. And the detailed mechanism of USP5 with different functions in GBM and how to identify the USP5-related GBM need to be further investigated.

In pancreatic carcinoma cells, knockdown of USP5 exhibited growth inhibition effects by suppressing cell cycle G1 to S transition, which was mediated by downregulating the cell cycle regulators (Kaistha et al., 2017). However, the underlying mechanism of USP5 in regulating cell cycle regulators is still under investigation. Consistent with the previous studies, we found that knockdown of USP5 in GBM cell lines could significantly inhibit cell proliferation *in vitro* and *in vivo* via induced arrest of cell cycle G1 to S transition. Several proteins are critical for driving cell cycle G1 to S transition, including CDK2, CDK4, CDK6, CyclinD1, and CyclinE1. Here, we revealed that among these proteins, knockdown of UP5 suppressed CyclinD1 protein level without any alteration of its mRNA level. This phenomenon is consistent with the regulation of USP5 as a deubiquitinase, which leading us to continue the following studies. Proteins modified with k48-linked polyubiquitination are subsequently degraded in proteasome (Swatek and Komander, 2016). In our study, decreased CyclinD1 protein caused by knockdown of USP5 could be rescued by treatment of proteasome inhibitor MG-132. Moreover, overexpression of USP5 obviously prolonged the half-life of CyclinD1 protein. Furthermore, USP5 was found to contact with CyclinD1 and decreased k48-linked polyubiquitination level on CyclinD1 protein. These results suggested that USP5 was essential for CyclinD1 protein stabilization.

It had been reported that USP5 could be activated by multiple stimuli, such as heat stress (Xie et al., 2018) and nociceptive information (Stemkowski et al., 2017; Joksimovic et al., 2018). Previous study had revealed that USP5 was ubiquitinated and degraded by Smurf1 through the proteasome pathway (Qian et al., 2016). Moreover, recent investigations had found that smurf1 functioned as an oncoprotein *via* mediating PTEN ubiquitylation in GBM(Chang et al., 2018; Xia et al., 2020). Thus, further studies could focus on the mechanism of how USP5 is regulated in GBM.

A number of USPs play critical roles in different malignant tumors *via* regulating cell cycle progression (Kaistha et al., 2017; Kitamura and Hashimoto, 2021; Yuan et al., 2021) and are continuously investigated as potential therapeutic targets. Recently, a variety of chemical entities were developed as USPs inhibitors, such as Pimozide, GW7674, Trifluooerazine, Rottlerin for USP1(Chen et al., 2011), ML364, LCAHA, 6TG for USP2 (Davis et al., 2016; Magiera et al., 2017; Chuang et al., 2018), GEN-6640, GEN-6776, FT67, FT827 for USP7 (Kategaya et al., 2017; Turnbull et al., 2017). USP14 inhibitor VLX1570 was the first DUB inhibitor to enter clinical trials for multiple myeloma therapy, but was deferred due to pulmonary toxicity (Wang et al., 2015; Rowinsky et al., 2020). Excitingly, increasing numbers of USP5 inhibitors were under drug discovery, such as RA-9 (Issaenko and Amerik, 2012), Vialimin A (Okada et al., 2013), Curcusone D (Cao et al., 2014), and showed a broad prospect for molecular therapy.

Taken together, our findings revealed a novel critical role for USP5 in the maintenance of GBM proliferation *via* deubiquitinated and stabilized CyclinD1 to promote cell cycle progression. These results provided another potent molecular target for GBM clinical therapy, and further research could screen or develop clinically available chemicals for USP5 inhibition.

## Data Availability

The original contributions presented in the study are included in the article/supplementary material, further inquiries can be directed to the corresponding authors.
